# *C. elegans dkf-1* (Protein Kinase D1) mutants have age-dependent defects in locomotion and neuromuscular transmission

**DOI:** 10.17912/micropub.biology.000800

**Published:** 2023-04-04

**Authors:** James R. Johnson, Jeff W. Barclay

**Affiliations:** 1 Institute of Systems, Molecular and Integrative Biology, University of Liverpool, Liverpool, England, United Kingdom

## Abstract

Changes in neuronal function that occur with age are an area of increasing importance. A potential significant contributor to age-dependent decline may be alterations to neurotransmitter release. Protein kinases, such as Protein Kinase C and Protein Kinase A, are well characterised modulators of neuronal function and neurotransmission. Protein Kinase D (PRKD) is a serine/threonine kinase whose role in neurons is less well characterised. Here we report that mutations in the *C. elegans* PRKD homolog, *dkf-1* , show an acceleration in age-dependent decline of locomotion rate and an alteration to age-dependent changes in aldicarb sensitivity. These effects could be explained by a pre- or post-synaptic function of the protein kinase as the animal ages.

**Figure 1. Phenotypic analysis of dkf-1 mutants.**
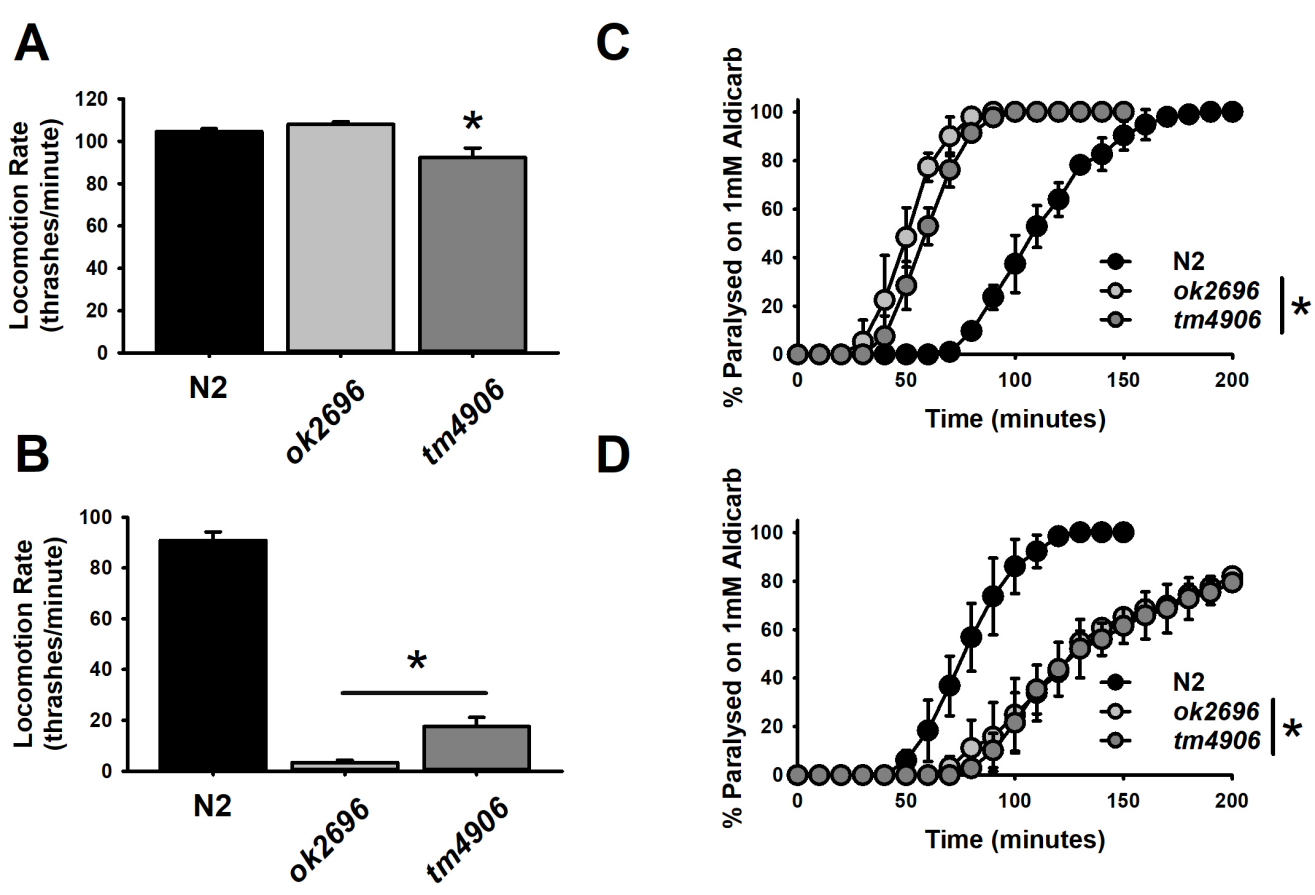
**A.** Thrashing rates of young (day 1 adulthood) Bristol N2 wild-types versus two independent *dkf-1* mutants ( *ok2696* , *tm4906* ). Only the *tm4906* strain showed a reduction in thrashing. **B.** Thrashing rates of older (day 5 adulthood) worms of the same strains. Both *dkf-1* strains showed a reduction in thrashing. **C.** Aldicarb sensitivity of young (day 1) Bristol N2 wild-types versus two independent *dkf-1* mutants. Both *dkf-1* strains were hypersensitive to aldicarb in comparison to wild-types **D.** Aldicarb sensitivity of older (day 5) worms of the same strains. Both *dkf-1* strains were resistant to aldicarb in comparison to wild-types.

## Description

Protein Kinase D1 (PRKD1) is a serine/threonine protein kinase associated with numerous cellular phenotypes including cell proliferation and survival, oxidative stress, trafficking and immunity (Zhang et al., 2021). As such, defects in PRKD1 lead to cancer, kidney disease, cardiovascular disease and inflammatory disease. PRKD1 is also expressed in neurons where it plays roles in development, survival, polarity and pain transmission (Li and Wang, 2014).

In addition to a serine/threonine kinase domain, structurally PRKD1 contains two upstream C1 domains that are thought to be activated by the lipid second messenger diacylglycerol (DAG) where it recruits the enzyme to the plasma membrane, in a similar manner to protein kinase C (PKC) (Zhang et al., 2021). In addition to DAG, PRKD1 also appears to require transphosphorylation by PKC to become activated. Once activated PRKD1 phosphorylates a host of downstream proteins (Franz-Wachtel et al., 2012).

Both of these upstream activation factors, DAG and PKC, are well known to alter synaptic signalling. DAG alters neurotransmitter release via direct interaction with the synaptic priming protein *unc-13* /Munc13 (Lackner et al., 1999, Rhee et al., 2002) and indirectly through the activation of PKC. In terms of synaptic signalling, the main characterised targets for PKC phosphorylation include SNAP-25 and Stxbp1/Munc18 (Barclay et al., 2003, Barclay et al., 2005). Despite its neuronal expression and its function downstream of both DAG and PKC, very little is known connecting PRKD1 to synaptic signalling.

In *Caenorhabditis elegans* , genetic mutations that increase levels of DAG or the addition of a pharmacological DAG analogue, the phorbol ester PMA, increase locomotion rate and make worms hypersensitive to aldicarb (Lackner et al., 1999, Nurrish et al., 1999). Conversely, *loss-of-function* mutations that block the downstream action of DAG or PMA, such as PKC ( *pkc-1* , *pkc-2* ), or expression of DAG-insensitive *unc-13* /Munc13 make worms resistant to the effects of aldicarb (Lackner et al., 1999, Sieburth et al., 2007, Edwards et al., 2012). Our understanding of the effects of PRKD1 on locomotion in *C. elegans* is limited and we know nothing of its effects on aldicarb strength.

In *C. elegans* , there are two PRKD1 isoforms, the best conserved of which is *dkf-1* . *dkf-1* is expressed in neurons around the nematode pharynx as well as in some tail neurons (Feng et al., 2006) whereas the other nematode isoform, *dkf-2* , is also neuronally expressed and contributes to lifespan and stress responses (Feng et al., 2007). Previously a locomotion defect has been reported for a *dkf-1* genetic mutation and following RNAi knockdown targeting *dkf-1* (Feng et al., 2006); however, the extent of locomotion defect was not explicitly quantified.

We therefore measured the locomotion rate directly by assessing thrashing in liquid of two *dkf-1* mutant alleles in comparison to Bristol N2 wild-type worms (Figure 1A). In contrast with previous reports (Feng et al., 2006), we found only marginal effects of the *dkf-1* mutations on locomotion rate when assessed at a young age (day 1 of adulthood – Figure 1A). The *dkf-1 ok2696* mutation had no effect on rate of thrashing, whereas the *tm4906* allele exhibited a small, but significant (~12%) decrease. A strong defect in locomotion, however, became evident with age. By day 5 of adulthood, an age-dependent decrease in locomotion for the Bristol N2 wild-type was minimal; however, there was a substantial, significant decrease in locomotion for both of the *dkf-1* mutant alleles (Figure 1B). The *ok2696* and the *tm4906* alleles had 96% and 81% age-dependent reductions in thrashing in comparison to only 13% for Bristol N2 wild-types.

*C. elegans* locomotion is impacted by effects on many different physiological properties, including neuromuscular signalling strength. Therefore, we assessed neurotransmission for our strains by aldicarb assay (Mahoney et al., 2006). Unlike mutations of PKC that cause worms to be resistant to inhibitors of cholinesterase (Sieburth et al., 2007, Edwards et al., 2012), surprisingly we found that either of the *dkf-1* mutations caused hypersensitivity to aldicarb at an early age (Figure 1C). Aldicarb sensitivity is known to increase with age in worms (Mulcahy et al., 2013) and we confirmed that we could quantify an increase in sensitivity for Bristol N2 at day 5 (Figure 1D). Strikingly, by day 5 both of the *dkf-1* mutant alleles demonstrated the opposite age-dependent effect to wild-types, becoming more resistant to aldicarb (Figure 1D).

Our results indicate that mutations in the PRKD1 orthologue *dkf-1* are associated with hypersensitivity to aldicarb at a young age, which is the opposite effect to that seen for PKC isoforms. This may be the result of an increase in endogenous neurotransmitter release. PRKD isoforms have been shown to phosphorylate a number of proteins that could potentially impact exocytosis, including those involved in post-Golgi vesicle trafficking and the cytoskeleton (Eisler et al., 2018, Weeber et al., 2019, Loza-Valdes et al., 2021, Grimaldi et al., 2022). Alternatively, although expression of *dkf-1* was not previously shown in body wall muscles, PRKD1 is expressed in muscle in humans where it interacts with the muscle protein titin (Herwig et al., 2020). Therefore, it is possible that *dkf-1* is also expressed in body wall muscle in *C. elegans* and that this expression contributes to a postsynaptic impact on aldicarb sensitivity. In support of this idea, overexpression of *dkf-1* did cause a decrease in nematode body length (Feng et al., 2006).

We have also identified some interesting age-dependent effects of *dkf-1* mutation. The almost complete reduction in thrashing rate and an inversion of aldicarb phenotype by day 5 of adulthood supports a progressive function for *dkf-1* . Indeed, PRKD1 is linked to neurodegeneration via a role in oxidative stress (Ay et al., 2015, Pose-Utrilla et al., 2017) and *dkf-2* worms have an altered lifespan (Feng et al., 2007).Nematode locomotion rate is known to decrease with age and specific mutations, such as cysteine string protein / *dnj-14* , can accelerate this decline (Kashyap et al., 2014). Mutations in many genes can alter aldicarb sensitivity in nematodes (Sieburth et al., 2005) and that sensitivity normally increases with age (Mulcahy et al., 2013). The switch from aldicarb hypersensitivity to resistance evident in *dkf-1* mutants implies a potential progressive effect on neuromuscular signalling or postsynaptic contraction during ageing. Future research will be essential to explore such age-dependent mechanisms in whole-animal models in more detail.

## Methods

*C. elegans* strains : All strains were cultured under standard conditions on Nematode Growth Medium agar plates at 20°C with *Escherichia coli* OP50 as a food source (Brenner, 1974). The following strains were used in this study: Bristol N2 (wild-type), RB2038 and FX04906. Both mutants have small (~400-500bp) N-terminal deletions of the *dkf-1* gene and were not backcrossed into Bristol N2 before phenotypic analysis. Only phenotypes observed in both independently derived alleles were considered specific to *dkf-1* function.

Phenotypic assays : All assays were performed on adult hermaphrodites at 20°C in a temperature-controlled room. Age synchronisation of worms was achieved by bleaching. During ageing, worms were picked to fresh Nematode Growth Medium agar plates daily to maintain age synchronisation of the worm population. Locomotion rate was quantified by thrashing in Dent’s solution (140 mM NaCl, 6 mM KCl, 1 mM CaCl2, 1 mM MgCl2, 5 mM HEPES, pH 7.4 with bovine serum albumin at 0.1 mg/ml) as described previously (Johnson et al., 2016, Johnson et al., 2017). One thrash was defined as one complete movement from maximum to minimum amplitude and back. Acute sensitivity to aldicarb was quantified by assessing the time to paralysis of a population of worms (Mahoney et al., 2006, Edwards et al., 2012). For each experiment, worms were placed on an unseeded Nematode Growth Medium agar plate supplemented with 1 mM aldicarb. Paralysis was determined by a failure to respond to a light mechanical stimulation assessed every 10 minutes following exposure to aldicarb.

Statistical analysis: All data are expressed as mean ± S.E. 20 worms per strain per age were used to assess locomotion. Significance for thrashing was assessed by one-way analysis of variance (ANOVA) with Tukey post-hoc test for multiple comparisons. For each aldicarb experiment, 25-30 worms per strain were used. Significance to aldicarb sensitivity was assessed by two-way analysis of variance (ANOVA) with Tukey post-hoc test for multiple comparisons. Experiments were performed three times.

## Reagents

**Table:** 

Strain	Genotype	Available from
Bristol N2	Wild-type *C. elegans*	CGC
RB2038	*dkf-1 (ok2696)*	CGC
FX04906	*dkf-1 (tm4906)*	Mitani lab
